# Three-Dimensional Patient-Derived *In Vitro* Sarcoma Models: Promising Tools for Improving Clinical Tumor Management

**DOI:** 10.3389/fonc.2017.00203

**Published:** 2017-09-11

**Authors:** Manuela Gaebler, Alessandra Silvestri, Johannes Haybaeck, Peter Reichardt, Caitlin D. Lowery, Louis F. Stancato, Gabriele Zybarth, Christian R. A. Regenbrecht

**Affiliations:** ^1^HELIOS Klinikum Berlin-Buch GmbH, Department of Interdisciplinary Oncology, Berlin, Germany; ^2^cpo – Cellular Phenomics & Oncology Berlin-Buch GmbH, Berlin, Germany; ^3^Medical Faculty, Department of Pathology, Otto-von-Guericke University Magdeburg, Magdeburg, Germany; ^4^Institute of Pathology, Medical University Graz, Graz, Austria; ^5^Eli Lilly and Company, Oncology Translational Research, Lilly Corporate Center, Indianapolis, IN, United States

**Keywords:** sarcoma, preclinical model, *in vitro* organoid culture, patient-derived *in vitro* model, drug screening, sarcoma treatment, personalized medicine

## Abstract

Over the past decade, the development of new targeted therapeutics directed against specific molecular pathways involved in tumor cell proliferation and survival has allowed an essential improvement in carcinoma treatment. Unfortunately, the scenario is different for sarcomas, a group of malignant neoplasms originating from mesenchymal cells, for which the main therapeutic approach still consists in the combination of surgery, chemotherapy, and radiation therapy. The lack of innovative approaches in sarcoma treatment stems from the high degree of heterogeneity of this tumor type, with more that 70 different histopathological subtypes, and the limited knowledge of the molecular drivers of tumor development and progression. Currently, molecular therapies are available mainly for the treatment of gastrointestinal stromal tumor, a soft-tissue malignancy characterized by an activating mutation of the tyrosine kinase KIT. Since the first application of this approach, a strong effort has been made to understand sarcoma molecular alterations that can be potential targets for therapy. The low incidence combined with the high level of histopathological heterogeneity makes the development of clinical trials for sarcomas very challenging. For this reason, preclinical studies are needed to better understand tumor biology with the aim to develop new targeted therapeutics. Currently, these studies are mainly based on *in vitro* testing, since cell lines, and in particular patient-derived models, represent a reliable and easy to handle tool for investigation. In the present review, we summarize the most important models currently available in the field, focusing in particular on the three-dimensional spheroid/organoid model. This innovative approach for studying tumor biology better represents tissue architecture and cell–cell as well as cell–microenvironment crosstalk, which are fundamental steps for tumor cell proliferation and survival.

## Introduction

Cancer is a group of diseases with a multitude of genomic aberrations typically classified by the cell of origin. Solid malignant neoplasms are predominantly carcinomas, which derive from epithelial cells, while a far less frequent group of solid neoplasms originates from mesenchymal cells. Normal mesenchymal cells form the soft and connective tissues as well as the bones. Tumors stemming from these cells are called sarcomas. They are malignant in most cases, and while their incidence in adults ranges from 1 to 2% (1–4), they account for up to 15% of all childhood and adolescence cancers (2, 3). Two main groups can be subdivided: soft-tissue sarcomas (STS) are more common in adults and represent 87% of all sarcomas, while sarcomas of the bone [osteosarcomas, Ewing sarcomas (EWS), and chondrosarcomas] occur more often below the age of 20 years (4, 5). Currently, the American cancer registry reports 4.2 cases per 100,000 for STS and 1.0 per 100,000 for sarcomas of the bone (6). Similar incidence rates have been reported for Europe (5, 7–9). Based on these numbers and according common definitions (10), sarcomas meet the criteria of rare diseases.

As for any rare disease, diagnostics and treatment should take place in specialized centers (7–9). Despite increased survival resulting from numerous multidisciplinary curative and palliative treatment options including surgery, monodrug or multidrug chemotherapy and/or targeted therapy, radiation therapy, hyperthermia, and isolated limb perfusion in a neoadjuvant or adjuvant setting (7–9), the disease outcome is often fatal. Currently, the 5-year relative survival rate for a patient with sarcoma considering the type, stage, localization, and age is about 60% (5) but dramatically dropping to 10% when only patients with advanced stages are considered (11). Due to the limited availability of tumor tissue for research and the complexity of the disease, progress in clinical management of sarcomas is lagging behind that of carcinomas. Since the lack of effective treatment options contributes to the low survival rate, the need for improving the treatment is evident.

### Risk Factors for Sarcoma Development

Sarcomas could stem from virtually any mesenchymal cell in the body, and new pathological and molecular methods used for tumor classification currently allow for the distinction of more than 70 histopathological subtypes (1, 2, 12, 13). This high degree of heterogeneity combined with low incidence makes systematic research of sarcomas scientifically challenging.

A large group of sarcomas develop spontaneously, but environmental and predisposing genomic factors have been found to increasing the risk of contracting this kind of tumor. For example, Kaposi sarcomas are known to be HIV or human herpes virus 8 induced (14). Common risk factors known to be causative for many malignancies such as exposure to certain environmental pollutants and chemicals, ionizing radiation (often in form of a previous radiotherapy), and inherited genetic aberrations are also confirmed to play a role in sarcomas (Table 1). Sarcomas can be classified based on their genomics into genetically simple and genetically complex sarcomas (15, 16). Sarcomas of the genetically simple category (hypomutated) are characterized by only one disease-specific “driver” aberration such as a translocation or mutation (Table 2) and are more common in younger patients. Most of the known translocations result in fusion genes which code for transcription or growth factors (15). Identifying these translocations is of great value to the pathologist, as they allow for a confirmed diagnosis where simple histopathology alone is not definite. For example, detecting the amplification of *MDM2* helps to confirm the diagnosis of a well-differentiated or dedifferentiated liposarcoma (17, 18). The genetically complex group (hypermutated) is made up by more or less chaotic karyotypes with high mutation frequencies in key oncogenes and tumor suppressor genes like *TP53* or *RB1* (15, 16). These complex genomic aberrations are commonly found in adult patients and/or as secondary lesions after radiation exposure (15) (Table 2).

**Table 1:** Common risk elevating factors for sarcoma development.

Risk factor		Resulting sarcoma subtype	Reference
Environmental pollutant/chemical	Ionizing radiation, previous or environmental	Especially osteosarcoma, angiosarcoma	(3, 5)
	Herbicides (e.g., phenoxyacetic acids, chlorophenol)	Non-specific	(5, 15)
	Vinyl chloride	Hepatic angiosarcoma	(15)
	Dioxins	Non-specific	(3)

Infection	HIV, human herpes virus 8	Kaposi’s sarcoma	(14)

Genetic disorder	Li–Fraumeni syndrome	Any cancer, 30% sarcomas, osteosarcoma and various soft-tissue sarcomas heaped among sarcomas	(3, 5, 12, 15)
	Neurofibromatosis type 1	Especially MPNST	(3, 4)
	Rb-mutation (13q14)	Especially osteosarcoma, if retinoblastoma has been survived	(3, 4)
	Paget disease	Osteosarcoma in adults	(5)
	Werner syndrome	Osteosarcoma	(4)
	Bloom syndrome	Osteosarcoma	(4)
	Gardner syndrome	Fibrosarcoma	(19)

*Shown are the most likely resulting sarcomas depending on risk factor but omitting carcinomas and other types of cancer even if they are more prevalent*.

**Table 2:** Common known aberrations of certain sarcoma subtypes.

Sarcoma subtype	Type of aberration	Locus	Reference
Gastrointestinal stromal tumors	Mutation	*cKIT* (exon 9 or 11) or *PDGFR-alpha*	(13)

Liposarcoma, well differentiated, and dedifferentiated	Amplification	*MDM2* (suppressor of p53)	(17, 18)

Myxoid liposarcoma	Translocation	FUS–DDIT3 [t(12:16)(q13;p11)]	(15)
		EWSR1–DDIT3 [t(12;22)(q13;q12)]	(15)

Alveolar rhabdomyosarcoma	Translocation	PAX3–FOXO1A [t(2:13)(q35:q14)]	(15)
		PAX7–FOXO1A [t(1:13)(p36:q14)]	(15)

Synovial sarcoma	Translocation	SS18-SSX [t(X;18)(p11:q11)]	(3, 15)

Ewing sarcoma	Translocation	EWSR1-FLI1 [t(11:22)(q24;q12)]	(15)
		EWSR1–ERG [t(21;22)(q22;q12)]	(15)
		EWSR1–ETV1 [t(7;22)(p22;q12)]	(15)
		EWSR1–ETV4 [t(17;22)(q21;q12)]	(15)
		EWSR1–FEV [t(2;22)(q33;q12)]	(15)

Myxoid chondrosarcoma	Translocation	EWSR1–NR4A3 [t(9;22)(q22-31;q11-12)]	(15)

Moreover, there are ongoing discussions about other potential risk factors for sarcoma development. Congenital or acquired immunodeficiency and, interestingly, also hernias seem to have suggestive evidence (3, 4). While often a trauma is reported in the patients’ medical history, publication showed that there is no such causative link between injury and sarcoma development, except for fibrosarcoma, dermatofibrosarcoma, and for patients with Gardner’s syndrome who underwent surgery (19).

### Gastrointestinal Stromal Tumors (GISTs): Model for Developing New Targeted Therapies in Sarcoma

Gastrointestinal stromal tumors represent approximately 18% of all sarcomas and are the most common mesenchymal neoplasms of the gastrointestinal tract. Historically, GISTs have a poor prognosis with tumor recurrence within 5 years after complete resection in up to 50% of patients. An important improvement in the management of this neoplasm was achieved in 1998 due to the discovery of oncogenic mutations in the tyrosine kinase KIT (20). The subsequent development and exploitation of kinase inhibitors that specifically downregulate this aberrant signal transduction pathway improved GIST patient outcome (21, 22) and made this approach a model for treating sarcoma by targeting altered intracellular signaling molecules. In some cases, a specific treatment can now be selected to target a mutation in a molecular pathway if a drug targeting this pathway is available, even if this drug was originally approved for a different tumor type. An important example is imatinib, a kinase inhibitor originally approved for the treatment of patients with BCR-ABL-positive chronic myeloid leukemia, which is also a very effective inhibitor of KIT and thus showed increased efficacy in KIT-mutated GIST. However, further investigation of mutation status in GIST has revealed a specific mutation (PDGFRA D842V) that according to current guidelines mostly prohibits the use of imatinib (9) as patients with this mutation harbor a primary resistance to this drug (21).

### Cells of Origin of Sarcoma

**Figure 1:**
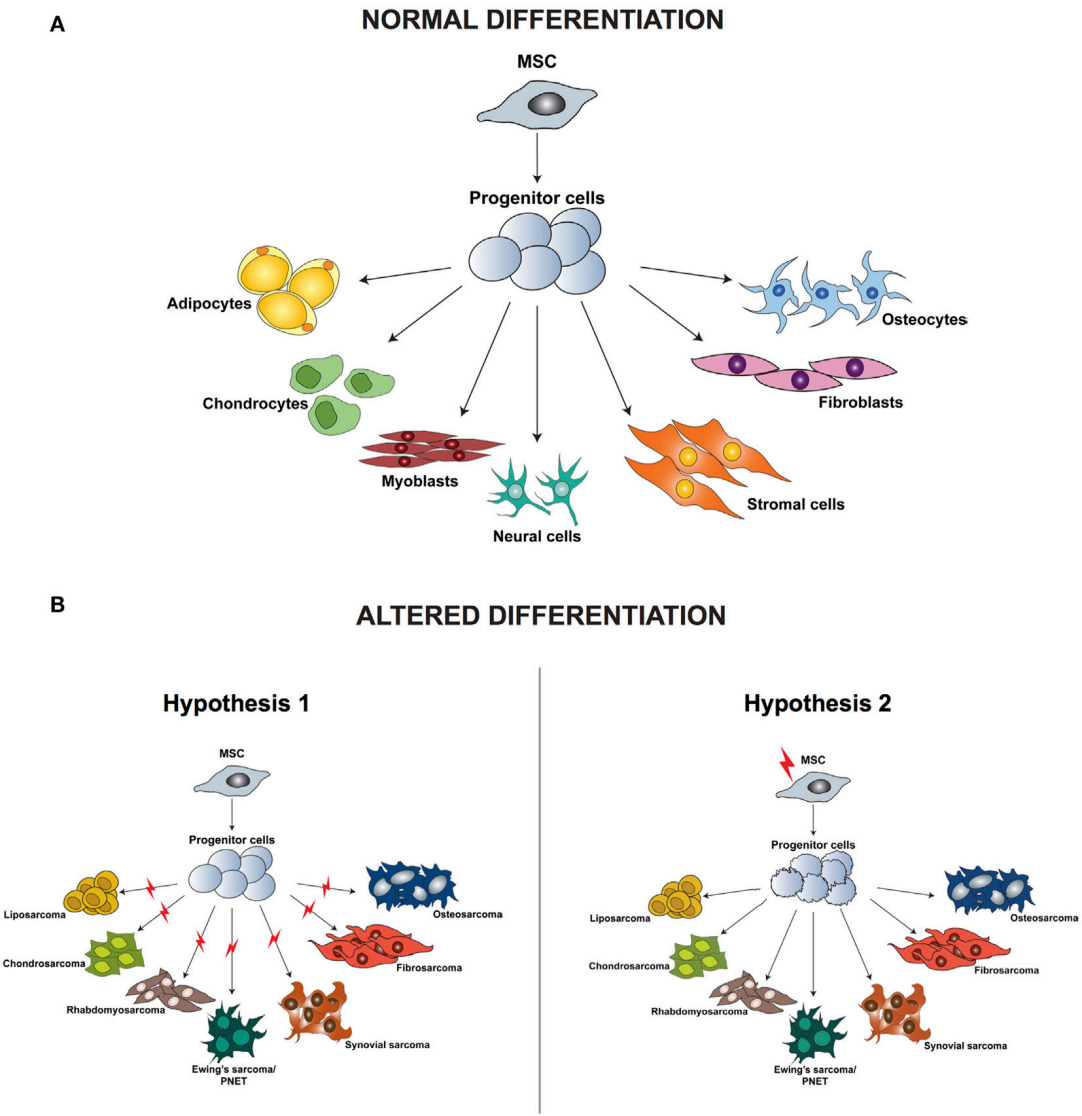
Differentiation of normal mesenchymal stem cells **(A)** and altered differentiation **(B)**. **(B)** The difference between the two hypotheses, whereby the initiating aberration occurs either at a later stage of differentiation (hypothesis 1) or hits the stem cell (hypothesis 2). Modified from the study by Teicher (24).

Irrespective of the clinical characteristics and in contrast to carcinomas, which arise from epithelial cells and are well defined by their tissue of origin, sarcomas are a group of highly heterogeneous tumors and evidence suggest that they develop directly from mesenchymal stem cells (MSCs) (23). MSCs are multipotent precursor cells of mesenchymal tissues such as bone, cartilage, fat, and muscle; several studies indicate their involvement in sarcomagenesis. Based on the wide variety of sarcoma subtypes, the origin of these tumors can be explained by two different hypotheses: the development of malignant alterations in a committed cell, distinct for every sarcoma subtype, or the presence of a common multipotent cell of origin that after transformation can differentiate into specific lineages (Figure 1).

According to the first hypothesis, tumors with a distinct phenotype and grade develop based on the basis of the lineage and of the differentiation stage when the initiating mutation occurs. This hypothesis is supported by studies in which the comparison between gene expression profiles of sarcomas and tissue-specific differentiation stages of MSCs showed a signature overlap in tumor and normal tissue according to their lineage of differentiation (25–29). One of the limitations of these analyses is the fact that they are based on *in vitro* cell culturing, which is known to induce alterations in the gene expression profile, thereby introducing a bias in the results. Moreover, it has been demonstrated that cells of a specific sarcoma subtype can differentiate into multiple lineages *in vitro* when specific inducing factors are added, thus indicating that not only the cell of origin but also the tumor microenvironment is fundamental for determining the final tumor phenotype.

Increasing evidence indicates that sarcomagenesis might be initiated by an aberration in a multipotent cell, and this hypothesis is currently favored by most researchers in the field. Several studies have demonstrated that mouse and human transformed MSCs can give rise to sarcomas after transplantation into mice. Miura and coworkers (30) showed that murine bone marrow-derived mesenchymal stem cells (BMMSCs) undergo spontaneous malignant transformation after prolonged culture (passage 29–54). Moreover, when injected in mice, these cells can form fibrosarcomas.

To test if MSCs are able not only to develop tumors when injected in mice after transformation but also to directly transform *in vivo*, Li et al. (31) transplanted bone marrow or MSC from male C57BL/6J mice into transgenic mice expressing a non-mammalian beta-gal enzyme (ROSA), chicken h-actin-enhanced GFP, and into WT littermates with bone marrow or MSC from male C57BL/6J mice as a control. After 18–24 months from transplantation, fibrosarcomas were the most common tumor detected, and immunohistochemistry analysis demonstrated that these tumors were derived from the transplanted bone marrow.

Compared to murine cells, human BMMSCs showed senescence without immortalization indicating that human MSCs cannot spontaneously transform (30); therefore, to translate the results obtained in mouse models, transformation of human MSC prior to inoculation is required. Genetic approaches aimed to knock out tumor suppressor genes and overexpress specific oncogenes have been used to induce MSCs transformation. The most common way to transform normal cells into malignant counterparts is the endogenous expression of human telomerase reverse transcriptase, simian virus 40 large T antigen (SV40-LT), and oncogenic H-RAS (32–34). Li et al. (35) applied this approach to study the origin of osteosarcoma. By using hMSC, they established cell lines by serially introducing these genetic alterations, and the effect of this manipulation on cellular phenotype, gene expression profiles, karyotype, and multilineage differentiation capacity was compared to osteosarcoma. They showed that two distinct genotypic and phenotypic sarcoma cell lines developed from these genetic events and that the transformed cells were characterized by increased motility. Moreover, transformed cells could be induced, so that osteogenic, adipogenic, and chondrogenic differentiation occurred, demonstrating that their multilineage differentiation potential was maintained.

Other groups studied MSC as cell of origin of osteosarcoma. Mohseny et al. (36) deeply characterized murine MSCs, transformed MSCs, and derived osteosarcoma cells lines genetically, phenotypically, and functionally, as well as for mRNA and protein expression. They identified aneuploidization, translocation, and homozygous loss of the *cdkn2* region as the key mediators of MSC transformation. In a cohort of 88 osteosarcoma patients, they showed a correlation between CDKN2A/p16 protein expression and prognosis, thus linking murine MSC model to human osteosarcoma. The genetic alterations that were found in both the *in vitro* cultured tumorigenic MSCs and the derived mouse tumors demonstrated that osteosarcoma could originate from MSCs. Interestingly, the fact that these cells could differentiate *in vitro* to chondrocytes and adipocytes but were prone to form osteosarcomas *in vivo* reveals the importance of the tumor microenvironment in determining the final tumor phenotype.

Finally, it has been shown that a subgroup of these multipotent cells express not only mesenchymal markers but also stem cell markers such as OCT3/4, NANOG, and SOX (37, 38) and that they are associated with drug resistance and metastasis development (39, 40). Taken together, these data suggest that MSCs might be not only the sarcoma initiating cells but also, due to their stemness, the cells responsible for maintaining tumor growth.

### Requirement of New Preclinical *In Vitro* Models for Improving Sarcoma Outcome

Besides earlier detection by novel imaging techniques, the overall survival of sarcoma patients has not improved in the last 30 years. This is mainly due to the lack of understanding of the biological consequences of the genomic alterations involved in sarcomagenesis. Therefore, it is clear that a better understanding of human sarcoma tumorigenesis and metastasis is pivotal to improve the management of sarcoma patients in terms of new therapeutic targets and approaches. Since each sarcoma subtype is characterized by a low incidence, the development of clinical trials is challenging, and the results are often biased by the limited number of patients involved (16). These limitations related to the nature of sarcomas make interdisciplinary approaches indispensable and the development of reliable preclinical models for molecular analysis and research of potential targetable nodes a priority. Even with technologies such as next-generation sequencing finding their way into the field of pathology, only the detailed understanding of the biology of sarcomas will foster new insights and as consequence translate to more effective therapeutic regimens in the clinic.

Recently, the efficacy of molecular methods in improving sarcoma diagnosis was tested in a multicenter, prospective study. For this study, the diagnosis of 384 patients from 32 French sarcoma centers using histopathology exclusively versus a combination of histopathology plus molecular characterization was reevaluated. The authors reported that for 53 of the patients considered an improvement was obtained when the diagnosis made by an expert pathologist was revised according to molecular genetic testing (41). This underlines the importance of sarcoma molecular characterization and demonstrates that molecular testing could significantly increase diagnostic accuracy.

In recent years, it has been extensively demonstrated that malignant tumors are characterized by varying degrees of heterogeneity where not only the primary tumor but also the corresponding distant metastasis have distinct genetic profiles (42, 43). Considering this heterogeneity, searching for actionable mutations using only next-generation sequencing techniques may be very challenging, and the treatment of tumor cells with specific mutations by targeted therapy could select for specific subpopulations resistant to the initial therapy (44–46), making the combination of multiple drugs with different targets the most promising approach, aiming at the inhibition of tumor growth at multiple levels. For example, Patwardhan et al. reported that a selective c-Fms/KIT inhibitor in combination with an mTORC1 inhibitor could be more effective than the c-Fms/KIT inhibitor alone in reducing tumor growth in malignant peripheral nerve sheath tumors in cell lines and xenograft *in vivo* models (47). These recent findings in sarcoma biology have encouraged the sarcoma research community into developing new predictive models for improving sarcoma treatment.

## Two-Dimensional (2D) *In Vitro* Models

Preclinical and translational studies of tumor mutations and aberrations as well as validation of therapeutic targets are based mainly on *in vitro* testing. Currently, the number of sarcoma models available for functional testing is still very limited, with only 2% of commercially available cell lines derived from STS (48). Moreover, the cell lines available do not represent the diversity of sarcomas, but are limited to the most common groups like osteosarcoma, leiomyosarcoma, and rhabdomyosarcoma with a total lack of more rare subtypes such as alveolar soft part sarcoma.

Due to these limitations, several groups focused on the establishment of new sarcoma cell lines. More than three decades ago, Bruland and coworkers isolated primary cells from 11 primary and metastatic human sarcoma specimens by enzymatic dissociation (49). Since the general success rate of sarcoma cell isolation was limited, they developed an alternative procedure using a non-adherent cell cultivation method (cellular spheroids) to the classical monolayer culture. With this approach, they produced stable monolayer cultures in 5 of the 11 samples used. These cells formed colonies in clonogenic soft-agar assays and developed tumors upon subcutaneous injection into nude mice. In 2002, additional cell lines were established, the majority from lung metastatic specimens derived from different sarcoma subtypes (50). In this study, all 11 cell lines analyzed expressed VEGF and basic-FGF, and they grew in anchorage-independent conditions. Moreover, when injected intramuscularly, six of the cell lines tested formed tumors and five of these spontaneously developed lung metastases, thus demonstrating the retention of tumorigenic and metastatic potential of the original tumor.

Recently, Salawa and coworkers established (48) primary cell cultures from fresh soft-tissue sarcoma samples with a success rate of 70%. For the seven long-term cell cultures that remained proliferative for at least 3 years and for more that 60 passages, they confirmed that the genomic and phenotypic characteristics were comparable to the original tumors. Since it is well known that long-term culture affects cell molecular characteristics, they analyzed the loss of heterozygosity (LOH) highlighting an increase of LOH after ~40 passages, thus demonstrating the presence of a genomic evolution commonly observed in *in vitro* cell cultures. Interestingly, three of the seven cell lines isolated were derived from undifferentiated pleomorphic sarcomas (UPSs) and the other four were derived from high-grade subtypes, suggesting a correlation between aggressive clinical course and the potential of *in vitro* growth.

Since studies based on the use of cancer cell lines often showed conflicting results, the characterization of the *in vitro* models used is very important, so that the results achieved by different laboratories can be compared. In 2010, the EuroBoNeT consortium characterized a set of 36 commonly used bone tumor cell lines (51), including osteosarcoma, EWS, and chondrosarcoma. After DNA fingerprint analysis to exclude cross-contamination of tumor cell lines, they showed that clones derived from the same original cell line (in this case, HOS) showed some differences from the parental line, suggesting a genomic evolution of the clones used in the study. Moreover, they highlighted a discrepancy between *CDKN2A* homozygous deletions in osteosarcoma and EWS cell lines (42 and 36%, respectively) compared to primary sarcoma samples, in which the frequency of this deletion is expected to be lower. This observation suggests that the cell line panel analyzed may be enriched in more aggressive tumors that easily grow *in vitro*. Finally, they analyzed the expression of TP53, a marker accepted for response to chemotherapy. They reported that 7 of the 10 TP53wt osteosarcoma cell lines showed low levels of *TP53* mRNA transcripts and only weak or no staining for the corresponding protein. Moreover, this downregulation was present only in osteosarcoma cell lines but not in the other seven TP53wt bone tumor lines analyzed.

To select cell lines that are more representative of human osteosarcoma, the same research group further characterized the 19 osteosarcoma cell lines available in that study (52) by analyzing their ability to differentiate *in vitro* and their tumorigenic potential in nude mice. While the differentiation capacity toward osteoblasts, adipocytes, or chondrocytes was maintained in all cell lines with some cell lines able to differentiate in more than one lineage, only eight cell lines developed tumors after subcutaneous and intramuscular injection into nude mice. In mice injected with HOS-143B cell line, multiple lung metastasis was detected during autopsy, demonstrating the metastatic potential of these cells. Interestingly, the availability of the non-tumorigenic HOS parental line and the corresponding non-metastatic HOS-MNNG makes these lines an excellent model for studying osteosarcoma progression.

Since 2D *in vitro* models are inexpensive and relatively easy to generate and maintain, they have been broadly used in preclinical research. However, these models do not accurately recapitulate the three-dimensional (3D) structure of tumor tissues and the complex crosstalk between tumor cells and microenvironment.

## 3D *In Vitro* Models

Forcing cells to grow in 2D induces alterations in cell morphology that in turn translates in changes of the gene and protein expression, as well as cell behavior compared to the tissue of origin (53–55). These limitations are partially overcome by 3D cell cultures that represent the donor-tissues’ architecture including cell–cell and cell–matrix interactions and are thus valuable tools for investigating the influence of the microenvironment and gradients of nutrients and oxygen on the interplay of cells within a tumor and their response to drug treatment (56). Since little is known about the molecular biology of sarcomas including unknown contextual cross talk between signaling pathways and other components presumably including epigenetic modifications and regulatory RNA sequences, patient-derived sarcoma tumor models are desirable tools to fulfill the promises of personalized medicine.

Several reports mainly aimed at the study of the presence of cells with cancer stem cell characteristics and their role in sarcoma tumorigenesis, local relapse, metastasis, and therapy resistance were published demonstrating that sarcoma cells can grow in non-adherent conditions, forming 3D structures called spheroids (49, 57–59). In 2009, Fujii et al. showed that commercially available human sarcoma cell lines such as MG63 (osteosarcoma), HTB166 (EWS), and HT1080 (fibrosarcoma) are characterized by the ability to form sarcospheres with stem-like properties. Moreover, they showed that cells grown as spheroids were resistant to doxorubicin and cisplatin, drugs frequently used for sarcoma treatment (39).

In additional to immortalized cell lines, primary cells can also form sarcospheres when grown in non-adherent and serum-starved conditions. Salerno and coworkers demonstrated that isolated tumor spheres were tumorigenic after transplantation into mice and that the tumors formed recapitulated the corresponding human disease (58). In addition, they showed that by modification of cell culture conditions, it was possible to influence the growth of the sarcospheres. Mimicking the tumor microenvironment by reducing O_2_ conditions to 1% induced a significant increase in the number and the size of the spheres demonstrating that 3D sarcoma models are useful tools for studying sarcoma development due to their flexibility.

To better model morphology, growth kinetics, and protein expression profiles of human tumors, Fong et al. (59) established an *ex vivo* 3D model of EWS by culturing TC-71 cells in porous 3D electrospun poly(ε-caprolactone) scaffolds. After a 20-day culture, a well-differentiated EWS-like phenotype was preserved in this *in vitro* 3D model as verified by expression of diagnostic markers such as CD99, keratin, and smooth muscle actin. Considering that one of the most promising new treatment strategies in EWS is the inhibition of the IGF-1R/mTOR pathway, they showed that the activation of IGF-1R/mTOR signal was higher in the 3D model, compared to the 2D counterpart, suggesting that the 3D microenvironment has a more physiological effect on the intracellular signaling cascade. Finally, they tested the sensitivity of this 3D model to doxorubicin, a cytotoxic chemotherapeutic agent used in EWS treatment. Similar to the lower sensitivity observed in xenografts, an increased resistance was observed in the 3D model compared to 2D cells. Taken together, these data demonstrated that EWS 3D models are useful and reliable tools for evaluating new IGF-1R antagonists not only as single agents but also in combined strategies. Moreover, since they better mimic the tumor microenvironment, they provide important information for identifying new signaling nodes that can represent potential targets for therapeutic intervention.

One of the main characteristics of EWS cells is the recruitment and activation of osteoclasts, leading to the destruction of bone tissue by osteoclast-mediated osteolysis. As this process is crucial, several groups focused on the development of *in vitro* models of bone osteolysis by coculturing tumor cells with osteoclasts and osteoblasts. Recently, Villasante et al. (60) engineered a healthy bone tissue by co-culturing osteoblasts derived from hMSC and osteoclasts derived from monocytes isolated from blood samples. First, hMSC were seeded within a decellularized bone scaffold and differentiated toward osteoblasts. CD14^+^ monocytes were then cocultured with osteoblast and differentiated in osteoclasts. EWS aggregates were finally infused into the tissue-engineered bone and maintained in culture. The analysis of the bone microenvironment highlighted a decrease in bone density, connectivity, and matrix deposition in the presence of EWS cells. Moreover, the treatment with antiosteolytic drugs inhibited this process limiting osteoclast-mediated bone resorption. Taken together these data highlight the feasibility of developing bone-mimicking models for the study of bone tumors and bone metastasis development. The possibility of using patient-derived induced pluripotent stem cells for developing the bone niche suggests the potential of using this model for creating personalized models useful for precision medicine.

### 3D *In Vitro* Models: From Carcinoma to Sarcoma Research

**Figure 2:**
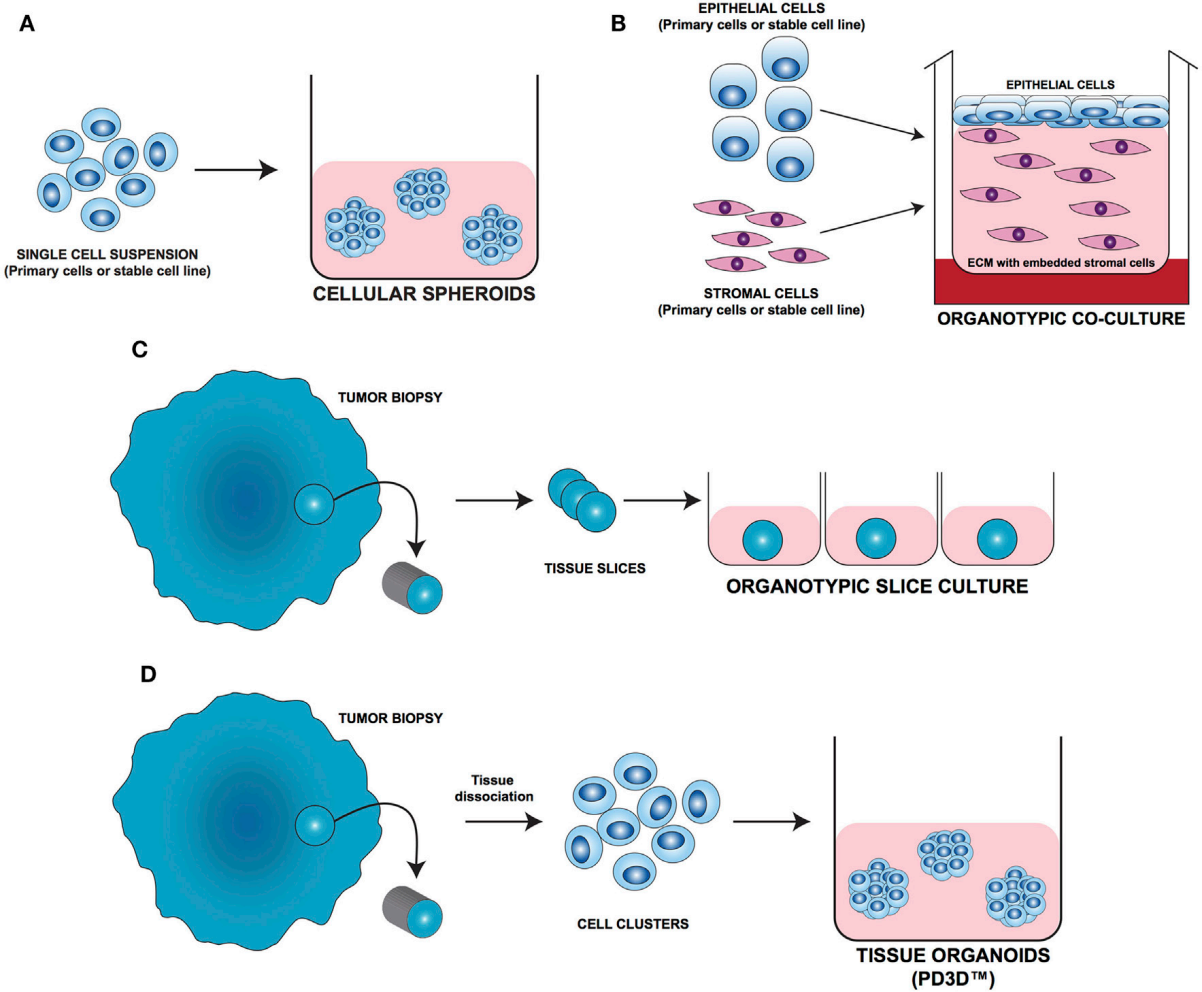
Different approaches for 3D cell culture model development. **(A)** Cellular spheroids: single cells from primary or stable cell lines aggregate together forming 3D structures. **(B)** Organotypic coculture: epithelial cells are cocultured with stroma cells embedded in a supporting matrix. **(C)** Organotypic slice culture: tissue slices obtained from the whole organ or from fragments of it are directly cultivated *ex vivo*. **(D)** Tissue organoids (PD3D™): primary cells isolated from fresh tissue without prior cell enrichment are grown as 3D multicellular structures [Modified from Silvestri et al. (64)].

**Figure 3:**
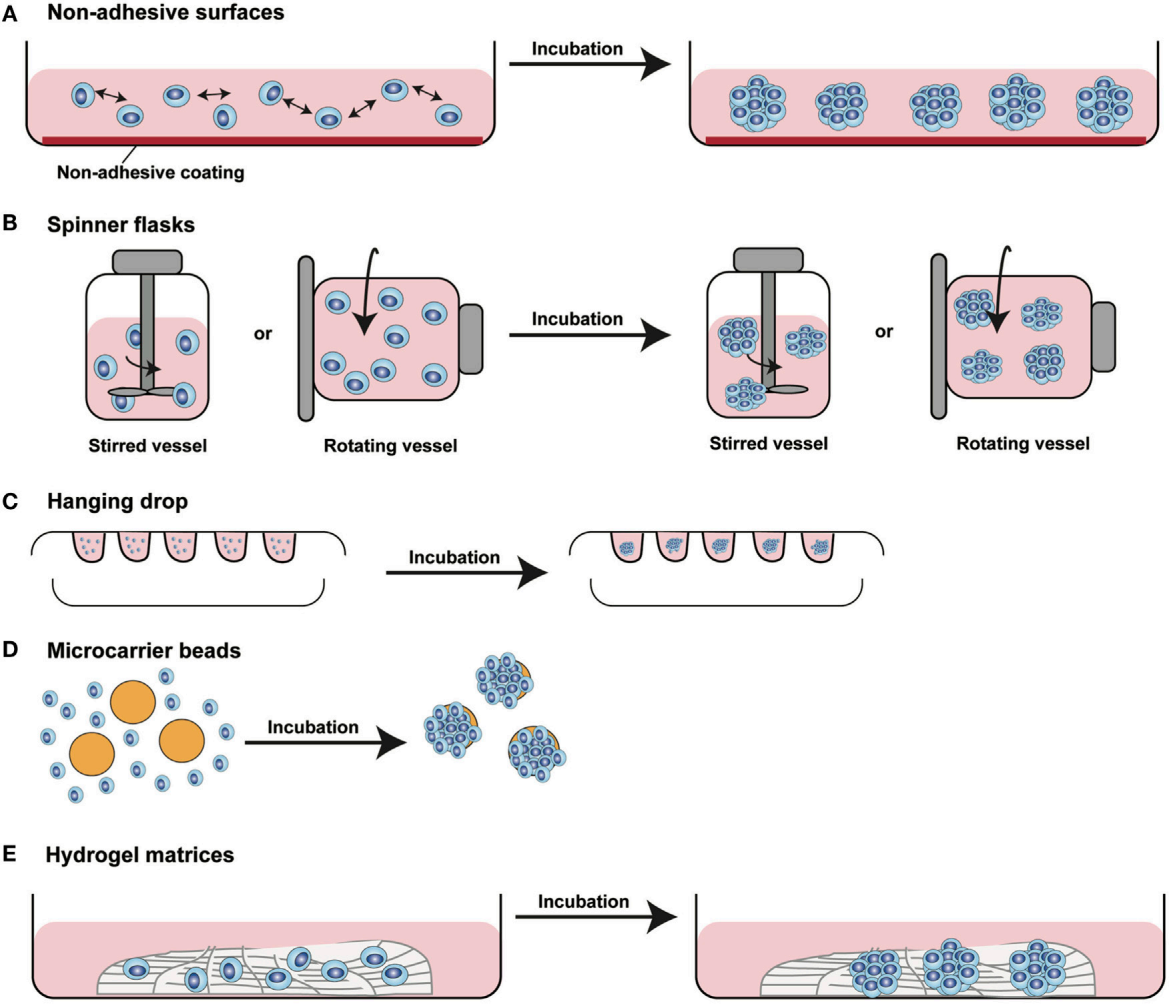
Different methods for 3D spheroids development and growth. **(A)** Non-adhesive surfaces: culture plates with modified surfaces to reduce cell adhesion stimulate cell aggregation and formation of 3D structures. **(B)** Spinner flasks: stirred or rotating vessels are used to prevent cell adhesion to the surface of the plate allowing 3D spheroids formation. **(C)** Hanging drop: cells seeded in small drops of medium form cellular aggregates at the tip of the drop due to gravity forces. **(D)** Microcarrier beads: cells adhere to and proliferate on the surface of natural or synthetic solid beads forming 3D structures. **(E)** Hydrogel matrices: cells are seeded into matrices of natural or synthetic origin forming 3D structures by single cells aggregation or by monoclonal cell growth. [Modified from Silvestri et al. (64)].

In the last decade, several 3D cell culture models have been developed to study different aspects of tumor biology and to test the efficacy of new anticancer molecules. While these approaches have mainly been established using carcinoma cells, with little effort, they can be also applied to the study of sarcoma biology. The least complex and therefore most frequently used models are based on spontaneous cell aggregation where the reduction of the adhesive forces to the surface of the culture plate allows cells to adhere spontaneously to each other forming cellular spheroids (Figure 2A). These models can be maintained either by using non-adhesive surfaces or spinner flasks and gyratory rotators, the use of hanging drop cultures, embedding of tumor cells in hydrogel matrices, or by the use of microcarrier beads and scaffolds. To avoid cell adhesion to the substrate surface, *non-adhesive surfaces* (Figure 3A) can be generated by using coatings such as agarose, polyHEMA, positively charged polystyrene, or proteoglycans (61–63). More recently, culture plates with modified surface chemistry have been developed allowing for “out-of-the-box”-ready technology and more reproducible growth of cellular spheroids. Besides the biological limitations, the main technical limitation of this method is the formation of spheroids with variable size and the inability to process upscaling.

The aforementioned limitations are partially overcome using *spinner flasks or gyratory rotator* (Figure 3B) systems, bioreactors that allow continuous mixing of medium or a constant rotatory movement of the flask, which prevents cell adhesion (65). These methods allow massive production of spheroids, therefore representing the method of choice for growing high amounts of homogeneous spheroids for downstream applications.

A technique often used is the so-called *hanging drop* method (Figure 3C) that makes use of gravity to stimulate cell aggregation. Cells in suspension are plated in small drops onto the underside of a plate lid that is then carefully inverted. Due to gravity, the cells accumulate in the tip of the drop, forming spheroidal aggregates (66). For those cells that do not spontaneously aggregate, systems that facilitate cell to cell interaction have been developed. *Microcarrier beads* (Figure 3D) are characterized by differences in size and composition. Surface coating allows adhesion and proliferation of cells consequently forming minispheroids that in turn aggregate one to each other, thus forming bigger spheroids (67). Another system to facilitate cell aggregation is the use of solid scaffolds with different porosity composed by natural or synthetic materials such as collagen, chitosan, or d,d,-l,l-polylactic acid. After seeding, cells can migrate along the surface, aggregate, and create 3D structures (68).

As tumor cells do not exist as isolated entities but rather are part of a complex microenvironment, natural or synthetic *hydrogel matrices* (Figure 3E) that mimic the *in vivo* tissue architecture can be used to grow tumor cells in 3D structures. The choice of a naturally or synthetically composed gel can be based on the aim of the analysis, ranging from single component hydrogels, i.e., laminin, collagen, and fibronectin to more complex ones such as Matrigel™ or Puramatrix™ (69, 70). Tumor cells can also be grown together with other tissue components such as stroma and epithelial cells in organotypic cocultures (Figure 2B). This more complex model allows to study the influence of tumor microenvironment on tumor development and progression as well as on drug sensitivity (71–74).

One of the most important tools in medical research is the model able to mimic the physiological situation in the closest way possible. Since they are commercially available and easy to handle, most of the basic and preclinical research in the oncological field was done using immortalized cell lines. On the downside, long-term *in vivo* culturing and the immortalization process often cause alterations in the molecular and phenotypic characteristics of these cells that can strongly differ from the cell of origin. To overcome these limitations, fresh tissue directly obtained during surgery has been used for isolating and cultivating tumor cells. One of the most straight forward methods is to cultivate fragments/slices of the tumor tissue as so-called *organotypic slice cultures* (Figure 2C). Several research groups use this method to study drug uptake, proliferation, and cell death, as well as for molecular characterization before and after treatment (75–77). The main advantage of this system is that the original tissue architecture is preserved, allowing the immediate study of the normal/altered physiology. Using tissue slices that can be maintained in culture for a short period of time only and without the opportunity of further expansion strongly limits the use of these models.

**Figure 4:**
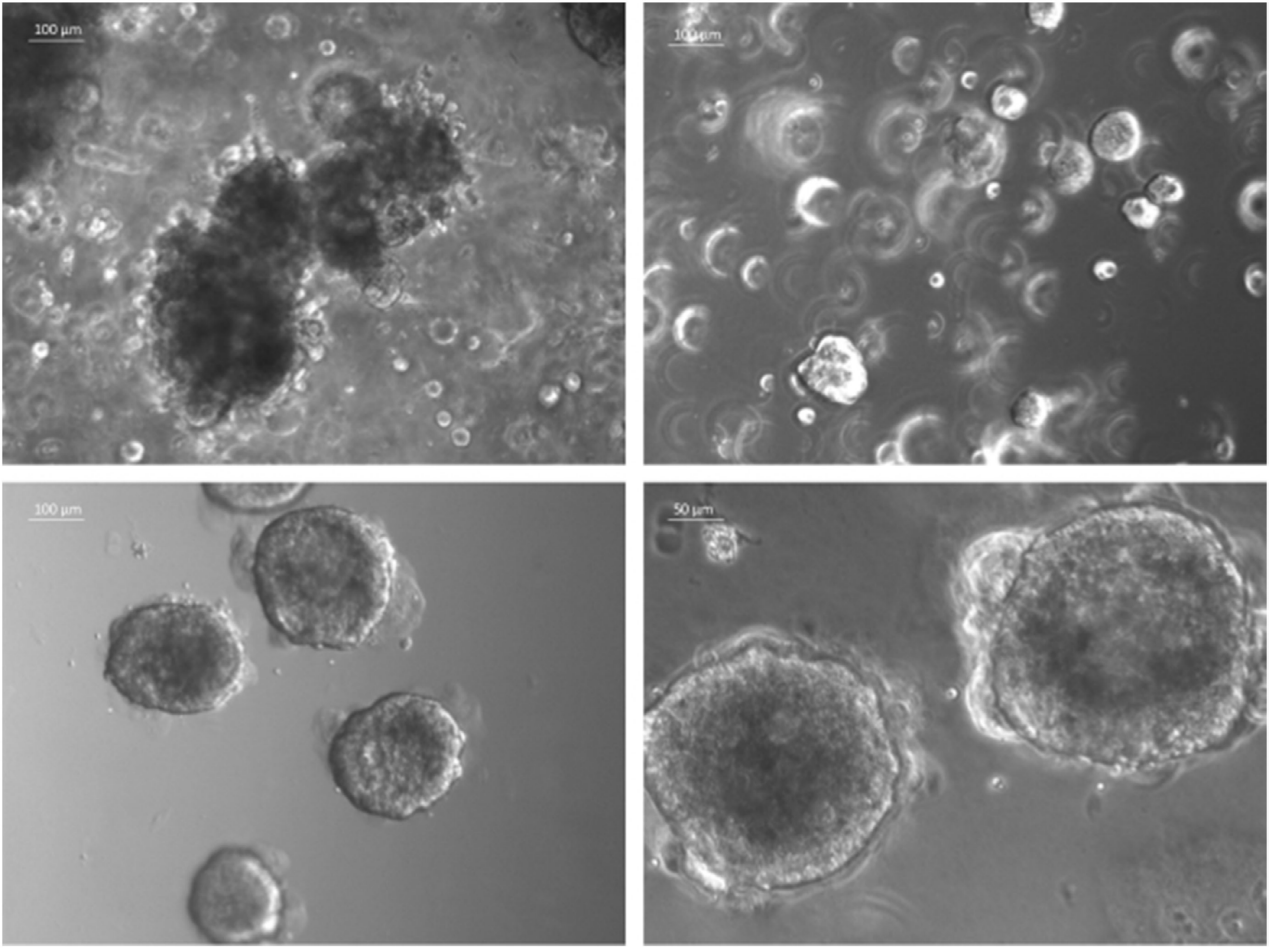
Sarcoma spheroids growing in Matrigel-based three-dimensional cell culture.

The currently most innovative and promising approach for *in vitro* model is represented by *tumor tissue organoids* (Figures 2D and 4). Organoids are multicellular structures directly isolated from primary tissue and grown in well-defined conditions. 3D organoids maintain the complex architecture of their tissue of origin and self-organize by reproducing their unique architecture and marker expression. This innovative tool has been used by several research groups for studying tumor development and progression and for testing drug efficacy (78–81). Interestingly, it has been recently demonstrated that this tumor model can be easily applied to high-throughput drug screening (82) and to correlate patient’s tumor molecular profiles to drug sensitivity (83).

## Molecular Drivers of Sarcoma Development as Novel Targets for Intervention

### Genomic Analysis of Driver Mutations

In 2010, Barretina and colleagues (84) performed an integrative system analysis of DNA sequence, copy number, and mRNA expression on 207 soft-tissue sarcoma samples including 7 major subtypes to identify novel subtype-specific genomic alterations representing potential therapeutic targets. They first studied genomic alterations in 47 tumor/normal DNA pairs highlighting 21 totally modified genes. These results were then validated in a second study-set of 160 tumors confirming the presence of subtype-specific mutations in several genes such as *PIK3CA* in myxoid/round cell liposarcoma, *TP53* in pleomorphic liposarcoma, and *NF1* in myxofibrosarcoma and pleomorphic liposarcoma. The data obtained are of high clinical potential since they helped identify tumors that might be responsive to PI3K or mTOR inhibitors, since NF1 loss causes mTOR pathway activation.

Recently, panel sequencing of 194 cancer-related genes in 25 STS was performed to identify actionable mutations (85). This analysis revealed the presence of different mutational profiles. In particular, in 60% of the cases targetable mutations for which clinical trials are available were highlighted while for another 28% of cases mutations which are currently not targetable were present. This study demonstrates the versatility of next-generation sequencing both in patient stratification for treatment with currently available therapeutics and in the identification of potential targets for developing new molecular treatments.

In the recent years, strong efforts have been made to find new biomarkers for selecting the best treatment based on specific tumor molecular profiles. With the goal of developing an efficient approach for patient stratification for treatment, Hanes and colleagues (86) combined tumor genomic characterization with drug testing *in vitro* in patient-derived cell lines. Three metastases from a patient with high-grade dedifferentiated liposarcoma previously treated with different chemotherapeutic agents were used for the study. Tumor tissues were analyzed by exome and transcriptome sequencing as well as DNA copy number analysis to highlight genomic aberrations that could represent potential targets for treatment. The data obtained were then used for selecting those drugs that can directly affect the altered gene or the corresponding signaling pathway in a cell line derived from the metastatic tissue. Among the altered genes observed in the tumor sample, an amplification of *FRS2*, the gene coding for fibroblast growth factor receptor substrate 2, was revealed. Based on this molecular alteration, they tested the *in vitro* efficacy of NVP-BGJ398 (infigratinib), a pan-FGFR inhibitor showing promising inhibition of proliferation induced by cell cycle arrest. Taken together, these data demonstrate the benefit of combining tumor genomic profiling with *in vitro* testing for a better selection of treatment in sarcoma patients and for selecting new promising treatments for improving sarcoma survival.

### Proteomic Analysis of Intracellular Pathways Alterations

Even if specific mutations have been associated with certain sarcoma subtypes, their etiology remain largely unknown. An equally important approach in biomarker discovery is the analysis of the proteome. Since the proteome is a functional translation of the genome, the information provided by its in-depth analysis may be a key in understanding sarcoma progression and therapy failure. Several research groups focused on differential expression of proteins in tumor tissue compared to the normal counterpart using diverse technologies such as Digiwest (87), 2D-PAGE (86, 87), mass spectrometry (87), and array technology (88).

Developing new sarcoma diagnostic biomarkers, Suehara and colleagues used 2D difference electrophoresis (2D-DIGE) analysis performing global protein expression analysis in different histological subtypes of soft-tissue sarcoma. Profiling data highlighted a set of 67 proteins distinguishing the 80 sarcoma samples based on their histological classification. Moreover, a signature of five proteins was able to differentiate at time of that publication known as grade III malignant fibrous histocytomas (today classified as UPS) and leiomyosarcomas into low- and high-risk groups characterized by significantly different survival rates (88).

The same research group applied a combined 2D-DIGE and mass spectrometry approach for profiling patients with GIST characterized by good and poor clinical prognosis (89), demonstrating the potential of this marker in GIST clinical management. This analysis highlighted 43 proteins (spots) differentially expressed and corresponding to 25 distinct gene products. Among these proteins, the authors focused on pfetin, a potassium channel protein, since 8 of the 43 spots that were found derived from this protein, and 4 of these had discriminative power between the two groups. Pfetin expression and its correlation with tumor metastasis was confirmed by real-time PCR and western blot. Moreover, the authors demonstrated that pfetin expression and 5-year metastasis-free survival rate were directly correlating.

In another recent study, 59 rhabdomyosarcoma samples were microdissected to enrich tumor cell content and analyzed by reverse phase protein microarrays (90), an antibody-based technology useful in studying the level of expression of selected total and phosphoproteins. This study showed that the phosphorylation of several components of the Akt/mTOR pathway was increased in tumors from patients with short-term survival. Moreover, an altered relationship between insulin receptor substrate 1, and this pathway was highlighted in patients with poor survival. The significance of these results was demonstrated by treating mouse xenografts with CCI-779, an mTOR inhibitor, that compared to controls greatly reduced the growth of two different rhabdomyosarcoma cell lines. These data showed the utility of phosphoproteomic pathway mapping for the study of functional drivers of sarcoma progression and for selecting patients for anti-mTOR/IRS therapy. These and other proteomic studies in different sarcoma subtypes were extensively reviewed by Kondo and colleagues (91).

Genomic and proteomic approaches can be synergistically applied for a deeper understanding of tumor biology at molecular level. Integrating these profiling systems, it is possible to correlate the presence of tumor-specific mutations to functional alterations in intracellular pathways. The information obtained from a multiomics approach may help in both designing new targeted therapies and selecting the best treatment option for a specific patient.

## Preclinical Drug Screening for Improving Sarcoma Treatment

Since no innovative therapeutic approaches are available for most sarcoma subtypes, several research groups focused on the discovery of new targets for sarcoma treatment by screening of compound libraries mainly on immortalized 2D cell lines and studying their effect on sarcoma cell biology.

As mentioned before, several sarcoma types are characterized by the presence of chromosomal translocations that cause the production of altered transcription factors. About 85% of EWSs express the EWS/FLI1 fusion protein, known to cause alterations in transcriptional regulation and RNA processing. EWS/FLI1 represents a very attractive drug target since it is specifically expressed by the tumor cells, but it is absent in the healthy tissue. Since, currently, no drugs targeting transcription factors are available, one approach is to directly or indirectly inhibit the players of the altered connected pathway. To this aim, Grohar and coworkers (92) screened more than 50,000 compounds in TC32 EWS cells for the ability of altering the expression level of the EWS/FLI1 downstream target NR0B1, that was prior transfected with a luciferase construct. The 200 compounds that showed activity in primary screening were further validated by multiplex PCR assay with the aim of selecting those hits that best inhibited the expression of a predetermined set of EWS/FLI1 downstream target genes. With this approach, they selected mithramycin as lead compound able to inhibit EWS/FLI1 activity. This effect was validated and further characterized in *in vitro* experiments and in *in vivo* xenograft models. Taken together, these data demonstrate the potential efficacy of this compound in treating EWS and the utility of applying high-throughput screening approaches for selecting new potential drug targets and new sarcoma therapies.

A similar study screened a small-molecule compound library containing FDA-approved drugs modulating the expression of EWS/FLI1 target genes on a panel of six EWS cell lines (93). To determine compound efficacy, the expression levels of few, well-characterized EWS/FLI1 target genes was measured. Among the 10 hits with the highest efficacy, several know therapeutic agents and fenretinide, currently in clinical trials for Ewing’s sarcoma, have been highlighted demonstrating the robustness of this screening approach. Moreover, midostaurin, a pan-kinase inhibitor, resulted in one of the most promising novel compounds. Interestingly, the efficacy of this drug was already shown in rhabdomyosarcoma, another pediatric sarcoma type. Moreover, midostaurin is currently undergoing phase II clinical trials for leukemia in adults and children, with a low toxicity in the pediatric population that make this drug a promising candidate to be tested in pediatric sarcoma patients.

The determination of new drug targets and efficient therapeutics requires even more the investigation of sarcoma subtypes that, unlike EWS, are not characterized by a known driver molecular alteration. Several groups tested available compound libraries to better characterize the drug sensitivity of different sarcoma subtypes and to correlate the response to specific compounds with the molecular characteristics of the tumor. Teicher and coworkers (94) screened the response of 63 sarcoma cell lines to 100 FDA-approved anticancer drugs and to a library of 345 investigational oncology agents. Moreover, they correlated treatment response with cell molecular profiles obtained by exon and microRNA arrays. The authors highlighted important correlations between cell characteristics such as sarcoma subtype and gene/miRNA expression, demonstrating that this screening approach is useful in studying the efficacy of FDA-approved drugs in specific sarcoma subtypes, in defining new potential therapeutic agents and for correlating sarcoma molecular profiles with drug sensitivity.

With such screening platforms available, it will become possible to investigate combination therapies for “vertical inhibition” of a single pathway or inhibition spanning multiple pathways.

## Clinical Opportunities for Patient-Derived 3D (PD3D) *In Vitro* Models

Ever since the sequencing of the first human genome, hopes were high that knowledge of the cancer genome landscape would bring an end to cancer and other diseases. Yet, sequencing alone has proven to be “remarkably unhelpful,” and the belief that sequencing your DNA is going to extend your life is “a cruel illusion” as James Watson put it in a recent interview with the New York Times (95). Today, genome researchers still struggle to be able to sufficiently support clinical decision-making with meaningful sequence data, and to compensate for this deficit, they propose that “more is more” (96). Yet, these genome centrics are neither feasible in the clinical setting nor payable by the majority of patients and insurance companies. Using PD3D cell cultures and exploiting their phenomics by combining multiple layers of evidence is expected to soon become the state-of-the-art approach. All current reports share the assumption that short-term PD3D cell cultures have already proven their superior predictive value in the preclinical arena, ousting other *in vitro* models in the development of new drugs. Pauli et al. have reported that they can successfully establish 3D cell cultures from surgically removed specimen within weeks (97). Therefore, leading comprehensive cancer centers around the world have started including patient-specific cell culture data in their infrastructure, as detailed by Shraddha Chakrandhar in Nature Medicine (98). Drug screenings in an automated setup, as described in 2016 by Boehnke et al. (82), take about 1 week once enough cells are available. In parallel, ultra-deep targeted sequencing can be performed, focusing on only those mutations that are relevant for the clinical decision-making.

Of course, the panels of target genes to be sequenced has to be updated with latest clinically relevant information to ensure that oncologists can stay focused on the immediate needs of the patients. Protein extracts from before and after the drug screening can be used for methods like Digiwest, a bead-based, multiplexed western blot (87). With this method, a selection of up to 200 (phospho-)proteins can be quantified at once, providing differential information not only on expression levels but also on the activation of key signaling kinases, such as those along them TOR, WNT, MAPK, or PI3K axes. Taking into consideration the time frame in which additional information can be used to support the decision-making process and the nature of information that becomes available from measuring cellular phenomics for the discussion in tumor boards, PD3D models may indeed become an integral part in clinical oncology of the 21st century.

## Conclusion

Recent research efforts in sarcoma has enabled important improvements in the knowledge of sarcoma histopathology that in turn defined sarcomas not as a single tumor entity but rather as different tumor subtypes with histology-specific molecular characteristics. The recent development of targeted therapies significantly contributed to the improved treatment options for sarcoma patients. Considering that this approach, first developed in carcinomas, showed efficacy in GIST, the next step in sarcoma research is to focus on the molecular characterization of the different subtypes to highlight new potential targets for therapy.

Since the availability of *in vitro* models that reliably represent the physiological tumor behavior is a prerequisite for successful sarcoma preclinical and translational research, several models have been developed for discovering new potential targets for therapeutic intervention. In particular, patient-derived cell lines and more recently 3D organoids represent innovative tools for studying molecular pathways promoting sarcoma development and tumor progression and for drug efficacy screenings. Moreover, these models could directly impact clinical decisions if and when used as a tool for precision medicine. 3D cultures directly isolated from a patient’s sarcoma could be used for testing the efficacy of drugs currently available, thus supporting clinicians in the selection of the most efficacious and promising treatment.

## Author Contributions

MG, AS, and CR drafted the original manuscript. CL provided the figures. JH, PR, LS, and GZ edited the draft and supported MG, AS, CR, and CL in finalizing the manuscript.

## Conflict of Interest Statement

No aspect of the submitted work has received payment or services from a third party. No entity has influenced the content of the submitted work. CR is founder and CEO of cpo. AS and GZ are employed at cpo. cpo is a contract research company working as a service provider in the field of 3D *in vitro* models and drug screening. CL and LS work in research at Eli Lilly and Company. MG and PR work at the Sarcoma Center at HELIOS Kliniken Berlin-Buch. HELIOS operates multiple hospitals in Germany and Europe. PR reports grants and personal fees from Novartis, personal fees from Pfizer, personal fees from Bayer, personal fees from PharmaMar, personal fees from Amgen, personal fees from GlaxoSmthKline, personal fees from Clinigen, personal fees from Lilly, and personal fees from AstraZeneca. None of them has been influencing the submitted work. All other authors declare no conflict of interest.
